# A novel nonsense mutation in the *STS* gene in a Pakistani family with X-linked recessive ichthyosis: including a very rare case of two homozygous female patients

**DOI:** 10.1186/s12881-020-0964-y

**Published:** 2020-01-31

**Authors:** Sibtain Afzal, Khushnooda Ramzan, Sajjad Ullah, Salma M. Wakil, Arshad Jamal, Sulman Basit, Ahmed Bilal Waqar

**Affiliations:** 1 Faculty of Allied and Health Sciences, Imperial College of Business Studies, Lahore, Pakistan; 2 Department of Genetics, King Faisal Specialist Hospital and Research Centre, PO Box 3354, Riyadh, 11211 Saudi Arabia; 3 Center for Genetics and Inherited Diseases, Taibah University, Madinah Al-Munawarah, Medina Saudi Arabia

**Keywords:** X-linked ichthyosis, Steroid sulfatase, *STS* gene, P,W96*, Pakistan, Affected females

## Abstract

**Background:**

X-linked ichthyosis (XLI; OMIM# 308100) is a recessive keratinization disorder characterized by the presence of dark brown, polygonal, adherent scales on different parts of the body surface. It almost exclusively affects males and the estimated prevalence ranges from 1:2000–6000 in males worldwide. Extracutaneous manifestations are frequent including corneal opacities, cryptorchidism, neuropsychiatric symptoms or others. Up to 90% of XLI cases are caused by recurrent hemizygous microdeletion encompassing entire *STS* gene on chromosome Xp22.3, while only a minority of patients shows partial deletions or loss of function point mutations in *STS*. Larger deletions also involving contiguous genes are identified in syndromic patients.

**Methods:**

Here, we report clinical and genetic findings of a large Pakistani family having 16 affected individuals including 2 females with XLI. Molecular karyotyping and direct DNA sequencing of coding region of the *STS* gene was performed.

**Results:**

The clinical manifestations in affected individuals involved generalized dryness and scaling of the skin with polygonal, dark scales of the skin on scalp, trunk, limbs, and neck while sparing face, palms and soles. There were no associated extra-cutaneous features such as short stature, cryptorchidism, photophobia, corneal opacities, male baldness, and behavioral, cognitive, or neurological phenotypes including intellectual disability, autism or attention deficit hyperactivity disorder. Molecular karyotyping was normal and no copy number variation was found. Sanger sequencing identified a novel hemizygous nonsense mutation (c.287G > A; p.W96*), in exon 4 of *STS* gene in all affected male individuals. In addition, two XLI affected females in the family were found to be homozygous for the identified variant.

**Conclusions:**

This study is useful for understanding the genetic basis of XLI in the patients studied, for extending the known mutational spectrum of *STS*, diagnosis of female carriers and for further application of mutation screening in the genetic counseling of this family.

## Background

Inherited ichthyoses are a heterogeneous group of skin disorders characterized by a defect of keratinization with the clinical appearance of scaling or hyperkeratosis or both. X-linked ichthyosis (XLI; OMIM #308100) is X-linked recessive skin disorder caused by a deficit in the steroid sulfatase enzyme (STS; EC 3.1.6.2). It has an estimated prevalence of 1:2000 to 1:6000 males worldwide; with no significant geographic or racial differences [1, 2]. Clinically, it is characterized by generalized dryness and scaling of skin in which the trunk, ears, neck, scalp and extremities are often involved; usually sparing palms and soles. Onset of symptoms is at birth or during the first months of life with the presence of mild-to-moderate dark, polygonal, adherent and regular scales of skin, more prominent on the extensor aspects of the limbs. XLI may occur solely as a skin disorder or it may be associated with several extracutaneous features such as corneal opacities, cryptorchidism, neuropsychiatric symptoms or others [3–5].

STS is a membrane-bound microsomal enzyme of 62 kDa capable of hydrolyzing 3-β-hydroxysteroid sulfates with ubiquitous expression in human tissues [6, 7]. In the skin, STS is expressed within the epidermis and play a role in steroid production and lipid metabolism. A deficiency of STS gives rise to excess accumulation of cholesterol sulfate in the stratum corneum that increases intercellular cohesion resulting in characteristic scale formation in XLI patients [8, 9]. The *STS* gene is located on distal short arm of X-chromosome (Xp22.3) region and spans more than 164 kb of genomic DNA. Almost ~ 90% of XLI patients harbor recurrent ∼2 Mb deletion of the entire *STS* gene and flanking sequences [1, 10–14]. The presence of variable nucleotide tandem repeat sequences on either side of the *STS* gene promotes mispairing at meiosis and unequal recombination, resulting in high frequency of these interstitial deletions observed in XLI patients [15].

Depending on the extent of the DNA deletion of Xp22.3, a patient may have XLI either isolated or also with other relevant phenotypical anomalies. Deletion of contiguous genes may result in syndromic conditions, e.g. Kallmann syndrome (OMIM #308700), X-linked recessive chondrodysplasia punctata (OMIM #302950), ocular albinism type I (OMIM #300500), and short stature (OMIM #300582) [1, 16, 17]. Partial deletions of the *STS* gene and point mutations are found only in a minority of patients [1, 11, 12, 18]. In the present study, we investigated a large Pakistani family with multiple affected members having clinical signs of XLI but without any detectable deletion in the *STS* gene. Instead a novel nonsense mutation (p.W96*) in *STS* gene was identified in the affected males in hemizygous state. Two females in the family exhibiting the diseased condition were also included and found to be homozygous for the mutation.

## Methods

### Patients

**Fig. 1:**
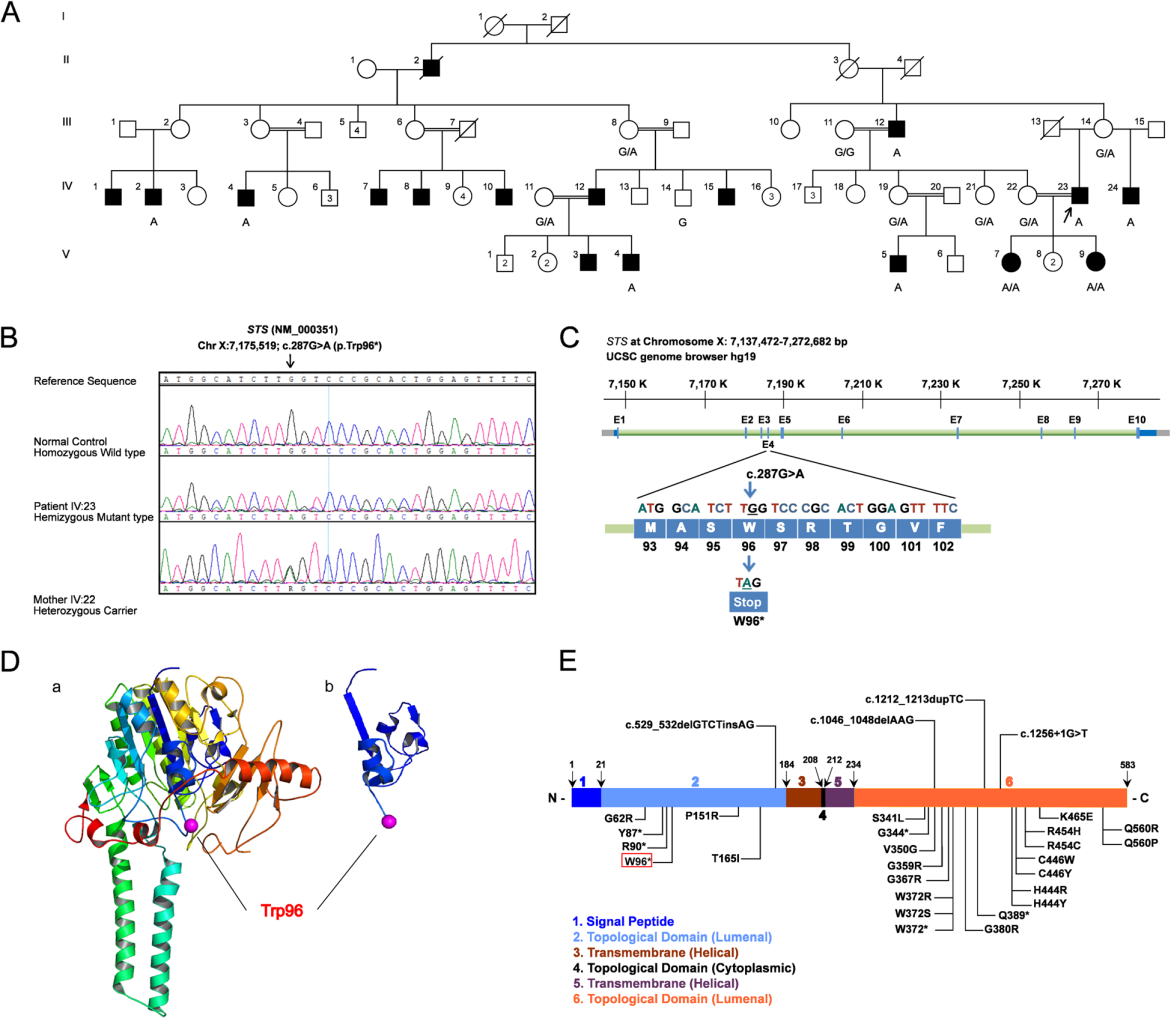
Identification of a novel nonsense mutation in *STS* gene (A) Pedigree of the family with X-linked ichthyosis. The proband (IV:23) is noted with an arrow. ■, affected male; ●, affected female; ◻, healthy male; ○, healthy female. (B) Sequencing chromatogram showing the wild type normal control hemizygous affected male and heterozygous female carrier, with mutation position marked by an arrow. (C) Schematic representation of exon-intron structure of *STS* gene; exons are designated as boxes E (1–10). DNA and protein sequence of the exon 4 where the mutation c.287G > A (p.W96*) resides is shown expanded. Nucleotide and amino acid numbering correspond to NM_000351 for the cDNA and NP_000342.2 for the protein. Nucleotides were numbered using A of the ATG translation initiation codon as + 1 nucleotide of the coding sequence. (D) A ribbon diagram showing the secondary and tertiary structures of the wild type STS enzyme. Sheets are drawn in yellow, helices in red and loop regions in green. (a) showing β-sheets, α-helices and coils. The structure (b) denotes the potential truncated polypeptide (STS enzyme) due to p.W96* mutation (Swiss-Model). (E) Schematic illustration of the domain graph of the encoded *STS* protein (Uniprot identifier: P08842), and the genetic variants. A full-length wild type STS protein is shown, with its N-terminus and C-terminus. Novel variant identified in our study is boxed in red alongside previously reported point mutations and a splice- site variant underlying XLI phenotype in The Human Gene Mutation Database (HGMD®; http://www.hgmd.cf.ac.uk)

A large five generation consanguineous family from village near Rawalpindi, Pakistan, was investigated in the present study (Fig. 1A). The family has 16 affected individuals including 14 males and 2 females. The pedigree analysis showed recessive X-linked mode of inheritance. Collection of family and personal history was based on a specific questionnaire (Additional file 1). Patients were seen by a dermatologist and neurologist. EDTA-anticoagulated peripheral blood (5 ml) was drawn from 17 family members (Fig. 1A). Written informed consent was obtained from all subjects or their respective guardians prior to their participation and the study was approved by the Ethics Review Committee of the Faculty of Allied & Health Sciences, Imperial College of Business Studies, Lahore. Following molecular diagnosis confirmation, patients performed physical examinations, ophthalmologic and neuropsychiatric evaluations.

### Molecular karyotyping/comparative genomic hybridization array (aCGH)

Genomic DNA was isolated from whole blood using Gentra Puregene Blood Kit (Qiagen, Germantown, MD), according to the manufacturer’s protocol. We used Cytoscan HD (Affymetrix, Santa Clara, USA) for molecular karyotyping which contains 2.6 million markers, of which 750,000 are genotyping single nucleotide polymorphisms and 1.9 million are non-polymorphic probes for genome coverage. The Cytoscan HD can detect copy number variation (CNV) and data analysis was performed using Chromosome Analysis Suite version Cyto2.0.0.195. Calling of pathogenic CNVs was in accordance with the ACMG (American College of Medical Genetics and Genomics) guidelines.

### Mutation detection

For mutational screening the ten coding exons and splice junction sites of *STS* gene were PCR amplified as described before [19]. Amplification by PCR was performed in a total volume of 25 μl, containing 20 ng DNA, 0.5 μM primers (Metabion, Germany), dNTPs at a final concentration of 0.2 mM, and HotStar Taq DNA Polymerase (Qiagen, Germantown, MD). PCR amplicons were sequenced using the Big Dye Terminator sequencing kit (Applied Biosystems [ABI], Foster City, CA). Samples were run on an ABI PRISM 3730 XL automated sequencer (ABI) and sequence analysis was performed using the SeqMan 6.1 module of the Lasergene (DNA Star Inc. WI, USA) software package and then compared to the reference GenBank sequence (accession number *STS*; NM_000351). To exclude the chances of neutral polymorphism a panel of 200 unrelated, unaffected, ethnically matched control individuals were screened. STS three-dimensional protein prediction models were viewed in a Swiss-Pdb Viewer, version 4.0.

## Results

### Clinical data

**Fig. 2:**
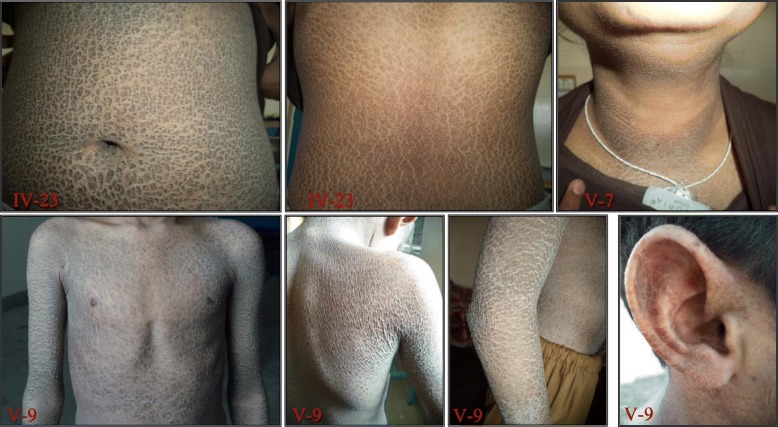
Clinical findings of XLI patients. Photographs of skin showing thick, large, polygonal, dark-brown scales involving the trunk front and back, neck, arm and ear

In all the patients from the family, the most prominent features included generalized dryness and dark brown scales prominent on the skin (Fig. 2); in which trunk, ears, scalp and limbs were affected. In almost all patients the abdomen was more severely affected than the back and other parts of the body. Scales were confluent and lighter on the scalp. Face, palms and soles were not involved. The hair and nails were normal. Preauricular small scales were also observed in young individuals. Their skin symptoms started by birth and during their youth and adolescence they were severely affected by this condition which consists of mild-to-moderate polygonal, dark brown, adherent and regular scales. The patients reported seasonal variation in disease severity. Their skin condition often exacerbated in cold weather but dramatically improves during the summer season, with the exception of very warm weather when insufficient sweating makes temperature regulation problematic with reduced heat tolerance. Physical examinations of the affected male members showed no micro-penises, cryptorchidism, small testes; ophthalmologic examination showed no corneal opacities or other any other abnormality. The growth, development and intellect of the affected members were normal. IQ of affected individuals was average in the range of 93 to 112.

### Molecular data

Initial genetic workup for two affected male patients (IV:4 and IV:23, Fig. 1A) involved molecular karyotyping, which was found to be normal. Direct Sanger sequencing of all the coding exons and splice sites of the *STS* gene revealed a hemizygous variant in exon 4 (chromosome X: 7,175,519; c.287G > A) in the index case (Fig. 1B). The identified variant was validated in all the included family members and all the affected males were found to be hemizygotes (Fig. 1A and B). The two affected females (V:7 and V:9, Fig. 1A) who were born to affected father (IV:23) and carrier mother (IV:22) in a consanguineous union, were found to be homozygous for the identified variant. The transition mutation c. 287G > A leads to a substitution of tryptophan at position 96 by a stop codon (p.W96*), producing a loss of 487 amino acids downstream (Fig. 1C). The sequence alteration was not found in 200 ethnically matched controls, dbSNP database build 151 (http://browser.1000genomes.org), 1000 Genome project and ExAC; discarding any possibility of polymorphism. In addition, the identified variant has not been described in the literature or Human Gene Mutation Database (HGMD®; http://www.hgmd.cf.ac.uk) as mutation. The identified variant p.W96* was predicted as “disease causing” by Mutation Taster. Hence the factors that p.W96* being a null variant, their absence in population data/controls, segregation analysis and relevance to the patients phenotype, led us to classify this allelic variants as “likely pathogenic” according to the recommendations of ACMG guidelines [20] for the interpretation of sequence variants. The identified variant in this study has been submitted to the ClinVar database at NCBI archives (ID: SUB5568796; (https://www.ncbi.nlm.nih.gov/clinvar/).

## Discussion

We performed molecular investigation of *STS* gene in a highly inbred Pakistani consanguineous family associated with XLI. The patients showed clinical features of dark brown scales especially prominent on the skin of the abdomen, legs and arms, segregated *STS* and a lack of any other phenotypic abnormalities. All the affected male members were found to carry a novel hemizygous mutation c.287G > A resulting in p.W96* premature termination of translation. Structural change predictions of the mutant form of STS with Swiss Model revealed that the p.W96* mutant is predicted to lack transmembrane, cytoplasmic and luminal domains thus resulting in loss of STS enzyme activity (Fig. 1D). Other possibility is the presence of non-sense-mediated mRNA decay (NMD) mechanism, which selectively degrades mRNAs harboring premature termination codons. However, in both instances it would result in absence or null activity of the STS enzyme.

Globally about 90% of XLI patients have complete deletions of the *STS* gene and flanking sequences, one of the highest ratios of chromosomal deletions among all genetic disorders [16, 21, 22]. A small number of point mutations with functional implications and partial deletions have also been reported (Human Gene Mutation Database, HGMD; (http://www.hgmd.cf.ac.uk, July 2019) without any evidence of genotype/phenotype correlation [18, 23]. So far, only 5 nonsense mutations, 2 in the N-terminal, 1 in the catalytic site and 2 in the C-terminal had been reported (Fig. 1E). The premature stop codon at codon 96 is expected to have serious consequences on the formation of mature protein and ultimately a deleterious effect on protein structure and function. This is consistent with other studies with nonsense hemizygous mutations detected in the sporadic subjects [24–27].

STS converts dehydroepiandrosterone sulfate in dehydroepiandrosterone, both steroids being involved in neurological processes [28]. Deletions encompassing *STS* and contiguous gene deletion syndromes have been associated with multiple behavioral, cognitive and neurological phenotypes notably: attention deficit hyperactivity disorder (ADHD), autism, mental retardation, and seizures [29, 30]. ADHD is also reported in XLI patients with partial *STS* deletions, suggesting that STS deficiency plays a direct role in the pathogenesis of inattentive or hyperactivity symptoms [31, 32]. None of our affected patients presented any neuropsychiatric symptoms; including ADHD, motor disabilities, behavioral abnormalities or social communication deficits. In addition, no other extra-cutaneous features or corneal opacities were detected in our family. The young family members have no learning disability and have normal academic performance in school.

The whole family is highly inbred and resides in a remote village in Pakistan; the elders and mothers are familiar with the disease and almost unmistakably notice the thin translucent membrane over the entire body for the affected newborns or a fine whitish scaling in the first days of life, confirming that XLI manifests around birth in a significant proportion of cases. However, dark polygonal scaling appears from late infancy to early childhood. The patients had not received any topical or systemic medical treatment, although the use of emollients helped with temporary remission of the cutaneous lesions. Clinical improvement during summer time is a common feature in ichthyosis patients. Family in our study belongs to a village in Pakistan and the male members work as farmers thus having a lot of sun-exposure, resulting in complete disease resolution during summer. Hypohidrosis is also known to be present due to keratotic plugs filling in sweat glands orifices [33]. The focus of treatment is lubrication, cutaneous hydration, and keratolysis, it should also include topical moisturizers and topical retinoids.

Our findings on lesion appearance and their distribution pattern in XLI patients are in keeping with literature survey [11, 33]. Scalp was affected in all the children and also mildly affected in few adults in the study family. The presence of fold involvement in about half of the reported cases limits its usefulness in differentiating XLI from ichthyosis vulgaris where folds are regularly spared. Because STS seems to play an important role in testosterone metabolism, the male-pattern baldness seems to be common in men with XLI [34]. Nevertheless, affected members of our family did not have any significant baldness pattern. Phenotypic variability of symptoms, even for patients with same *STS* mutations is likely due to effect of an individual’s genetic background, genetic modifiers and or other environmental factors. In addition the skin phenotype varies with age, older patients having dark brown scales than the younger individuals.

Recessive XLI is almost exclusively manifested in males. Female carriers of *STS* gene do not exhibit XLI symptoms, they may occasionally exhibit mild associated phenotypes such as dry skin [8, 23, 35, 36]. Interestingly, we observed no clinical signs of the any manifestations in the mothers of an affected male child, in agreement with previous reports and with the concept that *STS* gene is localized to a region of the X-chromosome that does not undergo X-inactivation [5, 16]. The presence of two XLI affected females with a homozygous *STS* mutation is an uncommon finding which could be explained by the high number of consanguineous unions in our family. So far, very few female patients have been reported in the literature [8, 37–42].

Diagnosis of XLI at or after birth is usually made clinically, but confirmation may be obtained through biochemically and the dissimilarities between XLI and regular skin and/or ichthyosis vulgaris can be quantified using protein electrophoresis [43]. Nevertheless, genetic diagnosis of affected individuals remains the most reliable method to identify the causative mutation leading to more accurate and effective genetic counseling, diagnosis of female carriers in the family allowing a correct evaluation of the risk of recurrence, especially in cultures where consanguineous marriages are preferred.

## Conclusions

In conclusion, we define a unique nonsense alteration in the *STS* gene which expands the spectrum of known *STS* mutations in XLI; although the molecular mechanism underlying pathogenesis requires further functional studies and research. We also report two affected female siblings from this highly consanguineous family; carrier testing for at-risk relatives in extended family members is offered for genetic counseling purposes.

## Supplementary information

**Additional file 1.** Questionnaire for recruitment of the patients with Ichthyosis for genetic studies.

## Data Availability

All data generated or analyzed during this study are included in this published article.

## Abbreviations

1000G: 1000 Genomes Project
ABI: Applied Biosystems
aCGH: Comparative Genomic Hybridization array
ACMG: American College of Medical Genetics and Genomics
ADHD: Attention Deficit Hyperactivity Disorder
CNV: Copy Number Variations
ExAC: The Exome Aggregation Consortium
HGMD: Human Gene Mutation Database
OMIM: Online Mendelenian Inheritance in Man
PCR: Polymerase Chain Reaction
*STS*: Steroid sulfatase
XLI: X-linked ichthyosis
