# Supplementary material for: A novel nonsense mutation in the STS gene in a Pakistani family with X-linked recessive ichthyosis: including a very rare case of two homozygous female patients
Source: BMC Med Genet. 2020 Jan 31;21:20. doi: 10.1186/s12881-020-0964-y (PMC6995215; doi:10.1186/s12881-020-0964-y)
Supplement: Supplementary file 1 — Additional file 1. Questionnaire for recruitment of the patients with Ichthyosis for genetic studies. [file 12881_2020_964_MOESM1_ESM.pdf]

Supplementary information: Additional file 1

Questionnaire for recruitment of the patients with Ichthyosis for genetic studies

Medical Record Number: ___________________________ Date: _______________________

Date of birth: _____________ Age at the time of disease onset: ________ Age at examination: _________

Sex (F/M): ____________

Please draw a family pedigree on the backside of this page

History of cutaneous disease in the family (Y/N): ____

If yes, what type of cutaneous diseases?____

History of any other genetic disease in the family (Y/N): ____

If yes, what type of genetic diseases? ____

Global severity of Ichthyosis ☐ Mild ☐ Moderate ☐ Severe ☐ Very severe

Dermatological Features ☐ Erythema ☐ Scaling ☐ Pruritus

Scale colour ☐ Brown ☐ Black

Scale Shape ☐ Polygonal ☐ Irregular

Cutaneous pain (Y/N): ______

Ocular troubles ___________________

Extra cutaneous findings

- ☐ Autistic
- ☐ Language delay
- ☐ Learning disability
- ☐ Behavioural anomalies
- ☐ Failure to thrive

Medications

Lubricating agents ☐ Petroleum/Vaseline ☐ Paraffin

Keratolytic agents ☐ Propylene glycol ☐ Salicylic acid ☐ Urea

Topical Retinoids

Other topical agents

Hydrating agents ☐ Glycerol ☐ Macrogol ☐ Propyleneglycol

Daily time spent for the skin care: ____

Any Additional Information: ____
